# The impact of a chemical reaction on the heat and mass transfer mechanisms in a dissipative and radiative nanofluid flow over a nonlinear stretching sheet

**DOI:** 10.1038/s41598-024-57952-9

**Published:** 2024-04-02

**Authors:** W. Abbas, Ahmed M. Megahed, Eman Fares

**Affiliations:** 1 Basic and Applied Science Department, College of Engineering and Technology, Arab Academy for Science, Technology and Maritime Transport, Cairo, Egypt; 2 Department of Mathematics, Faculty of Science, Benha University, Benha, Egypt; 3 Department of Basic Sciences, Faculty of Engineering, Pharos University, Alexandria, Egypt

**Keywords:** Nanofluid tangent hyperbolic model, Numerical treatment, Porous medium, Chemical reaction, Applied mathematics, Fluid dynamics

## Abstract

This paper presents a numerical investigation of the flow of a non-Newtonian tangent hyperbolic nanofluid over a nonlinearly stretched surface, taking into account factors such as thermal radiation, prescribed surface temperature, and a chemical reaction mechanism. Furthermore, the analysis includes the consideration of both viscous dissipation and the influence of a magnetic field within a Darcy porous medium. A mathematical framework for addressing the issue, rooted in the principles of conserving momentum, energy, and mass. The MATHEMATICA tools were employed to apply the shooting technique in order to solve the modeled equations describing the temperature, velocity, and concentration fields of the proposed physical system. Graphs are used to illustrate how certain key parameters affect the profiles of concentration, velocity, and temperature. Data tables are utilized to display information pertaining to the local Nusselt number, local Sherwood number, and local skin friction coefficient. The present results have been confirmed through a comparison with previously published findings. This research holds significant importance as it focuses on the extensive utilization of tangent hyperbolic nanofluids in cooling electronic components that produce substantial heat during their operation. The observed pattern indicates that as the local Weisbsenberg number, magnetic number, local porous parameter, and power law index increase, there is a reduction in the boundary layer thickness. Conversely, in the instances of concentration and temperature distributions, an escalation in these parameters leads to an expansion of the boundary layer thickness.

## Introduction

Over the past two decades, the study of non-Newtonian fluids has emerged as a highly consequential field of research. Consequently, there has been a substantial surge in interest surrounding the exploration of the thermophysical properties of these fluids. Given their extensive applications in various industrial and technological domains, a substantial body of literature has been dedicated to this subject, encompassing both analytical and numerical approaches to address this matter. Non-Newtonian fluids can be categorized into several models, including but not limited to the Williamson model^1^, Casson model^2^, Power-law model^3^, viscoelastic model^4^, Carreau model^5^, Maxwell type^6^, Casson and Williamson model^7^, Powell-Eyring model^8^, Cross model^9^ and more. Among non-Newtonian fluid models, the tangent hyperbolic fluid model^10^ stands out as a significant one. Within the field of chemical engineering, the tangent hyperbolic fluid model is an attractive substitute for the conventional Newtonian model. It has been demonstrated through laboratory experiments to accurately depict shear thinning behavior and effectively represents blood flow. Moreover, in various chemical engineering applications, this model offers distinct advantages over alternative non-Newtonian fluid models. Notably, substances like blood, ketchup, paint, nail polish, whipped cream, and similar materials exhibit tangent hyperbolic fluid characteristics in common practice. A wealth of fascinating research on the topic of tangent hyperbolic fluid can be accessed by referring to the citations numbered^11–13^. These references contain a diverse range of studies and investigations that delve into various aspects of tangent hyperbolic fluids, offering valuable insights and knowledge on this particular subject matter.

A nanofluid can be described as a mixture comprising a primary fluid, such as water^14^, combined with particles that are nanometer in size. The prevailing notion in this context is that the inclusion of nanoparticles is expected to enhance the overall heat transfer efficiency of these nanofluids. Because of their extensive application across various industrial systems, nanofluids have become a widely researched subject. They find utility in a diverse range of engineering systems for temperature regulation and the enhancement of operational efficiency. In 1995, Choi^15^ introduced the concept of nanofluids with the primary aim of enhancing heat transfer rates. Choi and Eastman^16^ were pioneers in the field when they initially proposed the notion that the incorporation of nanoparticles, ranging in size from 1 to 100 nanometers, into conventional base fluids serves to augment the thermal conductivity of these base fluids. The latest research findings^17–30^ have been consolidated to provide a comprehensive summary of the current state of knowledge on nanofluids.

While the preceding studies held significance and found application across various disciplines, their limitations were evident as they overlooked the influence of the viscous dissipation phenomenon coupled with variable thermal conductivity and chemical reactions within nanoparticles during the flow of nanofluid over a non-linear stretching sheet. Therefore, this research intends to investigate the behavior of a nanofluid model, specifically a tangent hyperbolic nanofluid, as it flows through a porous medium containing a nonlinearly stretching sheet. The study also considers the effects of thermal radiation, a magnetic field, and viscous dissipation in this context. The study also accounts for chemical reactions occurring among fluid particles and the varying thermal conductivity. As far as we are aware, no researcher has examined the model we are proposing up to this point. Hence, the incorporation of these phenomena in our research underscores the originality and novelty of our work. We utilize a similarity transformation to convert the governing partial differential equations, along with their corresponding dimensionless boundary conditions, into highly nonlinear ordinary differential equations. To numerically address the resulting nonlinear system of governing equations, we employ a shooting code for solving it. Charts and tables are utilized to analyze how various physical parameters influence the outcome. Finally, this type of nanofluid can find application in real life in manufacturing processes like metal cutting and forming, reducing thermal damage. Additionally, their heightened thermal conductivity makes them suitable for cooling systems in electronics and automotive applications, enhancing heat dissipation. Moreover, these nanofluids may be employed in solar thermal systems to improve the absorption and transfer of solar energy, thereby boosting the overall efficiency of solar collectors. We expect that this study will provide valuable insights and pave the way for further exploration of nonlinear stretching processes in an industrial and engineering context.

## Mathematical modeling

**Figure 1:**
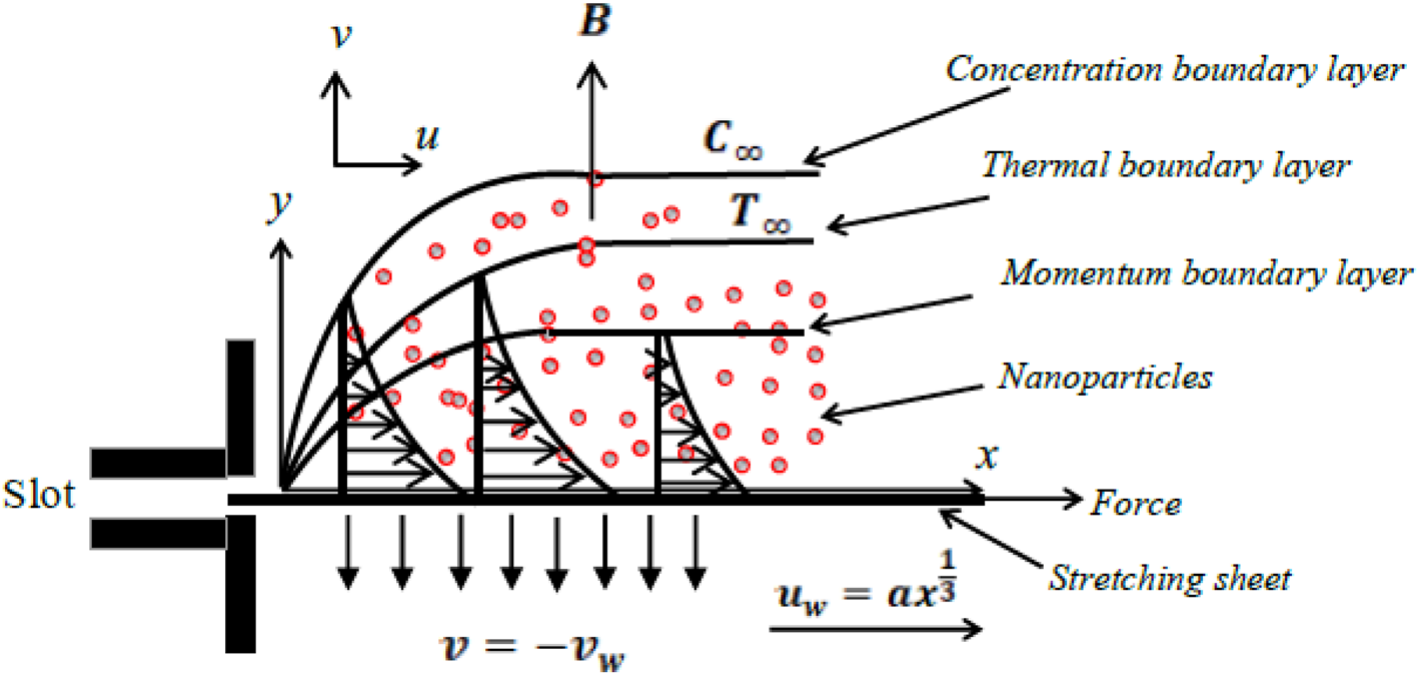
Geometry of nanofluid flow over a stretching sheet.

In this model, we consider the steady flow of a nanofluid with a hyperbolic tangent profile, taking into consideration its variable conductivity as it passes over a nonlinearly stretched sheet. The stretched sheet is oriented horizontally, with its length along the $x$-axis and the $y$-axis positioned perpendicular to it. The fluid’s movement is a result of the stretching of the horizontal sheet. Additionally, we make the assumption that the flow is confined to the region where *y* is greater than zero. The physical model is presented in a systematic manner in Fig. 1.

A constant magnetic field *B* in the transverse direction is applied along the $y$-axis, and the sheet is stretched in the $x$-axis direction with a velocity represented as $u_{w}=ax^{\frac{1}{3}}$. Also, the constant magnetic field *B* is assumed to vary with x coordinate in the form $B=B_{0}x^{\frac{-2}{3}}$. Also, $T_{w}, T_{\infty }$ and $C_{w}$ represent the temperature at the wall, the ambient temperature, and the concentration at the wall’s surface, respectively. We analyze the heat and mass transfer aspects, taking into account the influence of a chemical reaction phenomenon characterized by a conversion rate *K* and the presence of thermal radiation with a radiative flux denoted as $q_{r}$. The porous medium that encompasses the stretching sheet follows the fundamental principles of Darcy’s law, which govern fluid flow through porous materials. In the context presented here, the discussion takes into account thermophoresis effects and also investigates both viscous dissipation and the Brownian motion of nanoparticles that are suspended within the fluid. In their dimensional form, the governing equations, as described in references^31,32^, are expressed as follows:

1 $$\begin{aligned}{} & {} \frac{\partial u}{\partial x}+\frac{\partial v}{\partial y}=0, \end{aligned}$$

2 $$\begin{aligned}{} & {} u \frac{\partial u}{\partial x}+v\frac{\partial u}{\partial y}=\frac{\mu }{\rho }\left[ (1-n)\frac{\partial ^{2}u}{\partial y^{2}}+\sqrt{2}\,n\Gamma \,\frac{\partial u}{\partial y}\frac{\partial ^{2}u}{\partial y^{2}}\right] -\frac{\sigma B^{2}}{\rho } u-\frac{\mu }{\rho k}u, \end{aligned}$$

3 $$\begin{gathered} u\frac{{\partial T}}{{\partial x}} + v\frac{{\partial T}}{{\partial y}} = \tau \left[ {D_{B} \frac{{\partial C}}{{\partial y}}\frac{{\partial T}}{{\partial y}} + \frac{{D_{T} }}{{T_{\infty } }}\left( {\frac{{\partial T}}{{\partial y}}} \right)^{2} } \right] + \frac{\mu }{{\rho c_{p} }}\left[ {(1 - n)\left( {\frac{{\partial u}}{{\partial y}}} \right)^{2} + \frac{{n\Gamma }}{{\sqrt 2 }}\left( {\frac{{\partial u}}{{\partial y}}} \right)^{3} } \right] \hfill \\ \quad \quad \quad \quad \quad \quad \;\;\, + \frac{1}{{\rho c_{p} }}\frac{\partial }{{\partial y}}\left( {\kappa (T)\frac{{\partial T}}{{\partial y}}} \right) - \frac{1}{{\rho c_{p} }}\frac{{\partial q_{r} }}{{\partial y}}, \hfill \\ \end{gathered}$$

4 $$\begin{aligned}{} & {} u \frac{\partial C}{\partial x}+v \frac{\partial C}{\partial y}=D_{B} \frac{\partial ^{2} C}{\partial y^{2}}+\frac{D_{T}}{T_{\infty }}\frac{\partial ^{2} T}{\partial y^{2}}-K(C-C_{\infty }). \end{aligned}$$

Here, $u, v, c_{p}, \mu , D_{T}, D_{B}, C, \rho , \Gamma , n$ and $\tau$ correspond to the longitudinal and perpendicular velocity components, specific heat, kinematic viscosity, thermophoresis diffusion coefficient, Brownian diffusivity, concentration of nanoparticles, fluid density, time constant, power law index and the ratio between the heat capacity of the nanomaterial and that of the fluid. The applicable constraints or limits can be summarized as follows:

5 $$\begin{aligned}{} & {} v=-v_{w}, \quad u=u_{w}=ax^{\frac{1}{3}}, \quad T=T_{w}, \quad C=C_{w} \quad at \quad y=0, \end{aligned}$$

6 $$\begin{aligned}{} & {} u\rightarrow 0, \quad C\rightarrow C_{\infty }, \quad T\rightarrow T_{\infty } \quad \text{ as } \quad y \rightarrow \infty , \end{aligned}$$

where $v_{w}$ represents the velocity of suction which can be assumed to vary with *x* coordinate in the form $v_{w}=v_{0}x^{\frac{-1}{3}}$. The transformations that follow^33^ are introduced with the aim of converting the represented equations into a dimensionless form:

7 $$\begin{aligned}{} & {} \eta =\frac{1}{x^{\frac{1}{3}}}\sqrt{\frac{a}{\nu }}y, \quad u=ax^{\frac{1}{3}} f^{\prime }(\eta ), \quad v=-\frac{\sqrt{a\nu }}{3x^{\frac{1}{3}}} \left( 2f(\eta )-\eta f^{\prime }(\eta )\right) , \end{aligned}$$

8 $$\begin{aligned}{} & {} \phi (\eta )=\frac{C-C_{\infty }}{C_{w}-C_{\infty }}, \quad \theta (\eta )=\frac{T-T_{\infty }}{T_{w}-T_{\infty }}. \end{aligned}$$

In this context, the variable $q_{r}$ is streamlined and expressed as a function of temperature, which aligns with the prior research conducted by Alali and Megahed^25^. In this context, it is important to note that the applicability of linear radiation, based on a linear correlation between temperature and radiation heat transfer, is generally suitable for moderate temperature ranges. Consequently, our assumptions are aligned with scenarios characterized by moderate temperature distributions. Furthermore, we embrace the presumption that the thermal conductivity, denoted as $\kappa$, for the Tangent hyperbolic nanofluid varies as a function of dimensionless temperature $\theta$. This connection is elucidated by the subsequent equations^34^:

9 $$\begin{aligned} \kappa =\kappa _{\infty }(1+\varepsilon \theta ), \end{aligned}$$

where $\varepsilon$ represents the parameter governing thermal conductivity. After implementing the transformations mentioned previously in Eqs. (7)–(8), the governing equations take on the ensuing structure:

10 $$\begin{aligned}{} & {} \left( n\,\Gamma _{e}\,f^{\prime \prime }+(1-n)\right) f^{\prime \prime \prime }-\frac{f^{\prime 2}}{3}-(M+\beta ) f^{\prime }+\frac{2f f^{\prime \prime }}{3}=0, \end{aligned}$$

11 $$\begin{aligned}{} & {} \frac{1}{{\text {Pr}}}\left( (1+R+\varepsilon \,\theta )\theta ^{\prime \prime }+\varepsilon \,\theta '^{2}\right) +\delta _{b} \theta ^{\prime } \phi ^{\prime }+\delta _{t}\theta '^{2}+\frac{2f \theta ^{\prime }}{3}+Ec \left[ (1-n)f^{\prime \prime 2}+\frac{n \Gamma _{e}}{2} f^{\prime \prime 3}\right] =0, \end{aligned}$$

12 $$\begin{aligned}{} & {} \phi ^{\prime \prime }+\frac{2}{3}Sc f \phi ^{\prime }+\frac{\delta _{t}}{\delta _{b}} \theta ^{\prime \prime }-\gamma \phi =0. \end{aligned}$$

In addition to the following given boundary conditions:

13 $$\begin{aligned}{} & {} f=f_{w},\quad f^{\prime }=1,\quad \theta =1,\quad \phi =1, \quad at \quad \eta =0, \end{aligned}$$

14 $$\begin{aligned}{} & {} f'=0,\quad \theta =0,\quad \phi =0, \quad \hbox {as}\,\,\eta \rightarrow \infty . \end{aligned}$$

It’s evident that the resulting governing parameters are clearly defined as follows:

15 $$\begin{aligned}{} & {} \beta =\frac{\nu x^{\frac{2}{3}}}{a k }, \quad f_{w}=\frac{3v_{0}}{2\sqrt{a\nu }}, \quad M=\frac{\sigma B_{0}^{2}}{a \rho }, \quad \Gamma _{e}=\frac{\sqrt{2} a^{\frac{3}{2}}x\Gamma }{\sqrt{\nu }}, \quad Ec=\frac{u_{w}^{2}}{c_{p}(T_{w}-T_{\infty })}, \end{aligned}$$

16 $$\begin{aligned}{} & {} \delta _{t}=\frac{\tau D_{B}\left( C_{w}-C_{\infty }\right) }{\nu }, \quad R=\frac{16\sigma ^{*}T_{\infty }^{3}}{3\kappa k^{*}}, \quad \delta _{b}=\frac{\tau D_{B}\left( C_{w}-C_{\infty }\right) }{\nu }, \end{aligned}$$

17 $$\begin{aligned}{} & {} Sc=\frac{\nu }{D_{B}}, \quad \gamma =\frac{K x^{\frac{2}{3}}}{a}, \quad {\text {Pr}}=\frac{\mu c_{p}}{\kappa _{\infty }}, \end{aligned}$$

where $\beta$ is the local porous parameter, $f_{w}$ is the local suction factor, *M* is the local Hartmann number, $\Gamma _{e}$ is the local Weisbsenberg number, *Ec* is the local Eckert number, $\delta _{t}$ is the thermophoresis parameter, *R* is the radiation parameter, $\delta _{b}$ is the Brownian motion parameter, *Sc* is the Schmidt number, $\gamma$ is the chemical reaction parameter and ${\text {Pr}}$ is the Prandtl Number. Furthermore, we can mathematically express certain distinct physical factors to provide an intriguing insight into mass transfer, flow characteristics, and heat transfer mechanisms. These quantities are denoted as the local Sherwood number $Sh_{x}$, the local skin-friction coefficient $Cf_{x}$, and the local Nusselt number $Nu_{x}$. Their respective definitions are as follows:

18 $$\begin{aligned} Re_{x}^{\frac{-1}{2}} Sh_{x} =-\phi '(0), \quad Re_{x}^{\frac{1}{2}}Cf_{x}=-\left[ (1-n)f^{\prime \prime }(0)+\frac{n \Gamma _{e}}{2}f^{\prime \prime 2}(0) \right] , \quad Re_{x}^{\frac{-1}{2}} Nu_{x} =-\theta '(0), \end{aligned}$$

where $Re_{x}=\frac{u_{w}x}{\nu }$ is the local Reynolds number.

## Method of solution

Owing to the intricate nonlinearity inherent in Eq. (10) through (12), our present physical model does not possess an exact solution. Nevertheless, employing the shooting technique enabled us to convert these equations from boundary value problems to initial value problems. Subsequently, we successfully solved the system numerically by employing an appropriate initial guess. The difficulty encountered revolved around determining suitable initial values and conditions for the modified problem. In order to overcome this challenge, a transformation of the following is necessary:

19 $$\begin{aligned}{} & {} f=\chi _{1}, \quad f^{\prime }= \chi _{2}, \quad f^{\prime \prime }=\chi _{3}, \quad \end{aligned}$$

20 $$\begin{aligned}{} & {} \theta =\chi _{4}, \quad \theta ^{\prime }=\chi _{5},\quad \phi =\chi _{6}, \quad \phi ^{\prime }=\chi _{7}. \end{aligned}$$

In light of the preceding information, it is possible to deduce the following from the set of Eqs. (10)–(12):

21 $$\begin{aligned}{} & {} \chi _{1}^{\prime }=\chi _{2}, \quad \chi _{1}(0)=f_{w}, \end{aligned}$$

22 $$\begin{aligned}{} & {} \chi _{2}^{\prime }=\chi _{3},\quad \chi _{2}(0)=1, \end{aligned}$$

23 $$\begin{aligned}{} & {} \chi _{3}^{\prime }=\frac{\frac{\chi _{2}^{2}}{3}+(M+\beta )\chi _{2}-\frac{2}{3}\chi _{1}\chi _{3} }{n\Gamma _{e}\chi _{3}+(1-n)},\quad \chi _{3}(0)=\xi _{1}, \end{aligned}$$

24 $$\begin{aligned}{} & {} \chi _{4}^{\prime }=\chi _{5}, \quad \chi _{4}(0)=1, \end{aligned}$$

25 $$\begin{aligned}{} & {} \chi _{5}^{\prime }=\frac{-\varepsilon \chi _{5}^{2}+ {\text {Pr}}\left[ -\delta _{b}\chi _{5}\chi _{7}-\delta _{t}\chi _{5}^{2}-\frac{2}{3}\chi _{1}\chi _{5}-Ec\left( (1-n)\chi _{3}^{2}+\frac{n \Gamma _{e}}{2}\chi _{3}^{3}\right) \right] }{\left( 1+R+\varepsilon \chi _{4} \right) }, \quad \chi _{5}(0)=\xi _{2}, \end{aligned}$$

26 $$\begin{aligned}{} & {} \chi _{6}^{\prime }=\chi _{7},\quad \chi _{6}(0)=1, \end{aligned}$$

27 $$\begin{aligned}{} & {} \chi _{7}^{\prime }=-\frac{2}{3}Sc\chi _{1}\chi _{7}-\left( \frac{\delta _{t}}{\delta _{b}}\right) \chi _{5}^{\prime }+\gamma \chi _{6}, \quad \chi _{7}(0)=\xi _{3}, \end{aligned}$$

when $$\eta \rightarrow \infty$$

28 $$\begin{aligned} \chi _{2}\rightarrow 0,\quad \chi _{4}\rightarrow 0,\quad \chi _{6}\rightarrow 0. \end{aligned}$$

The iterative shooting method is utilized to seek appropriate estimates for $\xi _{1}, \xi _{2}$, and $\xi _{3}$, ensuring the fulfillment of outer boundary conditions $\chi _{2}(\infty )$, $\chi _{4}(\infty )$, and $\chi _{6}(\infty )$. This procedure can be reiterated until the target level of precision, specifically $10^{-6}$, is attained.

## Confirmation of the code’s precision

In this study, we utilized the shooting technique in conjunction with the Runge-Kutta scheme to conduct our numerical computations. The shooting method presents numerous advantages, such as its adaptability in addressing boundary value problems related to ordinary differential equations. It is versatile across a broad spectrum of problems and is easy to implement. Notably, the method demonstrates numerical efficiency and doesn’t necessitate a detailed formulation of the solution, enhancing its utility for intricate systems. To verify the effectiveness of the provided code, we obtained results for the skin friction coefficient $Re_{x}^{\frac{1}{2}}Cf_{x}$ at $\beta =f_{w}=0$ for various values of the power law index *n* while maintaining the other parameters at constant values, as illustrated in Table 1. The outcomes displayed in Table 1 indicate a strong concurrence between our findings and those acquired in the study conducted by Ahmed et al.^33^. This strong agreement serves to confirm both the precision and the reliability of our code and the numerical outcomes it produces. It is important to note that the originality and novelty of our proposed research, in comparison to the previous work by Ahmed et al.^33^, can be encapsulated by examining crucial phenomena such as viscous dissipation, variable thermal conductivity, and the chemical reaction process within a Darcy porous medium.

**Table 1:** Comparison table of $Re_{x}^{\frac{1}{2}}Cf_{x}$ with the previously findings of Ahmed et al.^33^ when $M=\Gamma _{e}=0.5$ and $\beta =f_{w}=0$.

*n*	Ahmed et al.^33^	Present work
0.1	0.932163	0.93216257890
0.2	1.016640	1.01663899187
0.3	1.096308	1.09630779902

## Discussion

**Figure 2:**
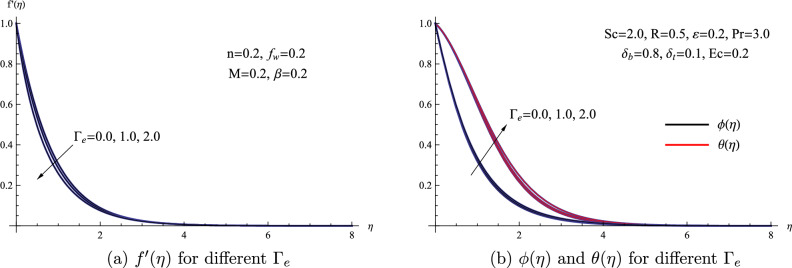
(**a**) $f'(\eta )$ for different $\Gamma _{e}$ (**b**) $\phi (\eta )$ and $\theta (\eta )$ for different $\Gamma _{e}$.

This portion of the study delves into the numerical analysis of how governing parameters affect the flow behavior of a magnetized Hyperbolic tangent nanofluid over a non-linear stretching sheet. The equations in this model are controlled by various key parameters that have recently emerged in the field. These parameters include the local porous parameter denoted as $\beta$, the local suction factor represented as $f_{w}$, the local Hartmann number indicated as *M*, the local Weisbsenberg number denoted as $\Gamma _{e}$, the local Eckert number labeled as *Ec*, the thermophoresis parameter referred to as $\delta _{t}$, the radiation parameter designated as *R*, the Brownian motion parameter represented as $\delta _{b}$, the Schmidt number termed as *Sc*, and the chemical reaction parameter known as $\gamma$. Figure 2 illustrates how the temperature $\theta (\eta )$, concentration $\phi (\eta )$, and velocity $f'(\eta )$ profiles change with respect to $\eta$ as the local Weisbsenberg number $\Gamma _{e}$ undergoes variations. The Weissenberg number $\Gamma _{e}$ is a dimensionless quantity that quantifies the relationship between the relaxation time of a material and the time scale associated with fluid flow. As depicted in Fig. 2, as $\Gamma _{e}$ increases, it becomes evident that a longer duration is required for the fluid to undergo its flow, leading to a reduction in velocity. Conversely, both temperature and concentration exhibit an opposite trend in response to the variations in $\Gamma _{e}$. The influence of $\Gamma _{e}$ on the characteristics of the flow of the nanofluid can be attributed to the fundamental concept behind $\Gamma _{e}$. Physically, the local Weisbsenberg number fundamentally assesses the speed at which the fluid moves in relation to the material’s capacity to deform or respond to external forces.

**Figure 3:**
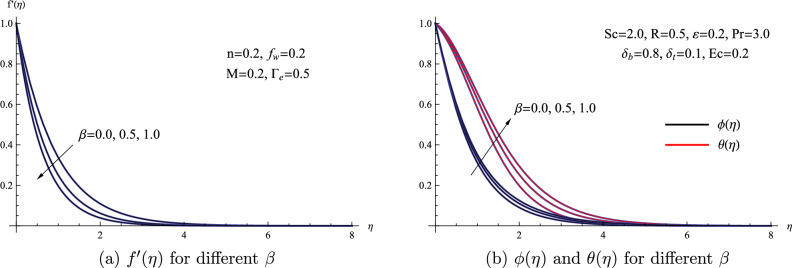
(**a**) $f'(\eta )$ for different $\beta$ (**b**) $\phi (\eta )$ and $\theta (\eta )$ for different $\beta$.

Figure 3 presents a visualization of the traits associated with the porous parameter $\beta$ and how it affects the flow properties of the nanofluid. A dimensionless quantity that deals with flows across porous medium is the porous parameter. It describes the porosity or permeability of a substance through which a fluid is flowing. This characteristic is crucial because it affects how easily and with what resistance fluid can pass through porous objects like geological formations, filters, and membranes. Hence, Fig. 3 effectively illustrates that as the porous parameter experiences an increase, there is a noteworthy reduction in the velocity of the nanofluid. Additionally, it is evident that the resistance of the fluid to the pores within the porous medium substantially amplifies both the temperature and concentration of the fluid. Physically, the decline in nanofluid velocity due to the porous parameter is mainly linked to the heightened resistance faced by the fluid when moving through the porous medium. This increased resistance acts as an obstacle to the fluid’s flow, leading to a decrease in velocity.

**Figure 4:**
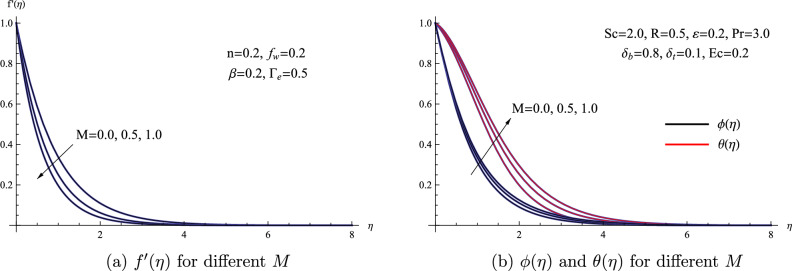
(**a**) $f'(\eta )$ for different *M* (**b**) $\phi (\eta )$ and $\theta (\eta )$ for different *M*.

Figure 4 provides a visual representation of how the Magnetic parameter *M* influences the characteristics of flow, heat, and mass within the nanofluid. It is evident that the fluid’s velocity experiences a reduction in the presence of a magnetic field. Furthermore, the same graph demonstrates that the presence of a magnetic force leads to an improvement in both the temperature $\theta (\eta )$ and concentration $\phi (\eta )$ distributions. In a physical sense, the Lorentz force exerted on the fluid increases with the rise in the magnetic parameter. Consequently, this causes a reduction in fluid velocity, ultimately slowing down the fluid’s movement. Moreover, the preceding research conducted by Yasmin and colleagues^35^ provides support and confirmation for the influence of the magnetic field on the heat transfer dynamics within nanofluid flow.

**Figure 5:**
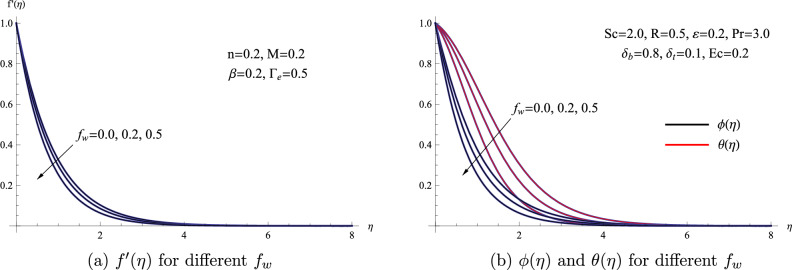
(**a**) $f'(\eta )$ for different $f_{w}$ (**b**) $\phi (\eta )$ and $\theta (\eta )$ for different $f_{w}$.

Figure 5 provides a graphical depiction of how changes in the suction parameter impact the characteristics of concentration $\phi (\eta )$, velocity $f'(\eta )$, and temperature $\theta (\eta )$ profiles. This graph demonstrates that with an increase in the suction parameter $f_{w}$, the concentration, temperature, and velocity profiles decline at elevated values. From a physical perspective, the reduction in nanofluid velocity with an increase in the suction parameter can be attributed to the presence of pores on the stretching sheet. These pores introduce resistance to the flow of fluid, leading to the observed decrease in velocity.

**Figure 6:**
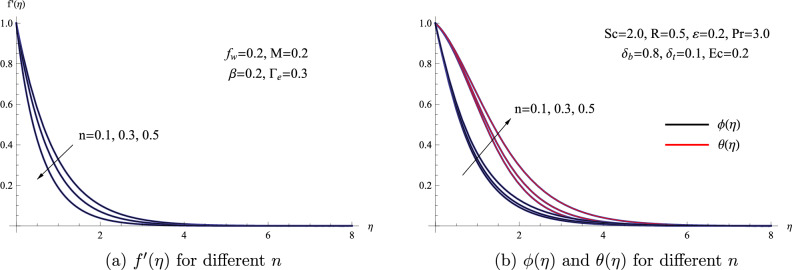
(**a**) $f'(\eta )$ for different *n* (**b**) $\phi (\eta )$ and $\theta (\eta )$ for different *n*.

Figure 6 illustrates the changes in the graphs of temperature, concentration, and velocity concerning variations in the power law index *n*. This visual representation showcases how these parameters evolve as the power law index undergoes alterations. It is quite apparent that there is a clear correlation between the increase in the power law index factor and the corresponding elevation of the fluid’s temperature, concentration, and the thickness of the thermal boundary layer. Furthermore, based on the information presented in the same figure, it becomes quite evident that when the power law index is intensified, there is a noticeable reduction in the velocity distribution within the boundary layer region. Physically, the reduction in nanofluid velocity associated with the power law index is mainly due to the fluid’s response to shear forces. A higher power law index indicates heightened resistance to shear, resulting in a decline in fluid velocity.

**Figure 7:**
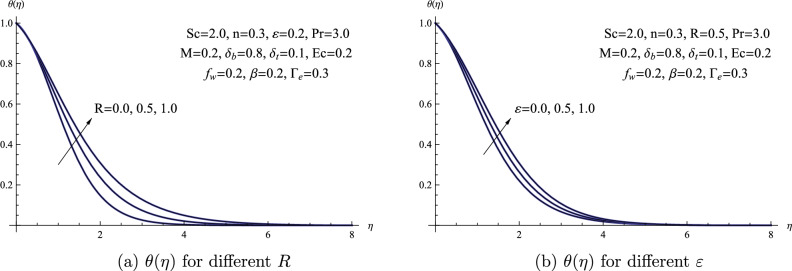
(**a**) $\theta (\eta )$ for different *R* (**b**) $\theta (\eta )$ for different $\varepsilon$.

Figure 7a provides a visual representation of how the thermal radiation parameter *R* influences the temperature profile $\theta (\eta )$. Essentially, the radiation parameter assists in assessing how significant thermal radiation is in a specific heat transfer situation, providing insight into whether it plays a prominent or inconsequential role. Consequently, with an increase in the radiation parameter, there is a notable upward trend in $\theta (\eta )$, resulting in the expansion of the thermal boundary-layer thickness. Additionally, previous studies^36–41^ provide confirmation of the influence of the thermal radiation parameter on the heat transfer mechanism. Figure 7b provides a depiction of how the temperature distribution, represented by $\theta (\eta )$, is influenced by changes in the variable thermal conductivity factor $\varepsilon$. As the thermal conductivity factor rises, the temperature of the nanofluid rises. Physically, this phenomenon happens as a result of a more effective heat transmission from the sheet to the nanofluid at higher levels of the thermal conductivity factor.

**Figure 8:**
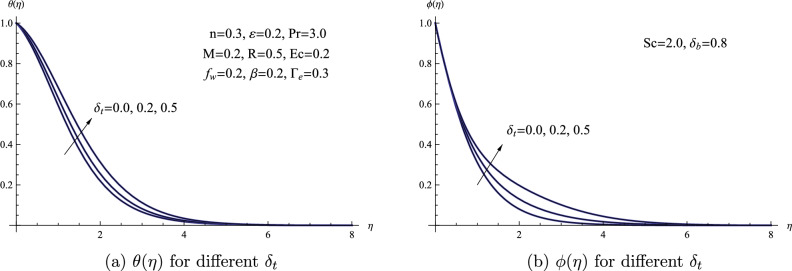
(**a**) $\theta (\eta )$ for different $\delta _{t}$ (**b**) $\phi (\eta )$ for different $\delta _{t}$.

Figure 8 visually demonstrates how variations in the thermophoretic parameter $\delta _{t}$ influence both temperature $\theta (\eta )$ and concentration $\phi (\eta )$. To begin, we introduce the concept of the thermophoretic parameter, a non-dimensional value relevant to thermophoresis. Thermophoresis is a phenomenon occurring in fluid dynamics and heat transfer where particles or molecules dispersed within a fluid experience motion due to temperature variations within the fluid. An escalation in the thermophoretic parameter, denoted as $\delta _{t}$, is depicted as leading to an increase in both temperature $\theta (\eta )$ and concentration $\phi (\eta )$ levels. Moreover, the rise in temperature and concentration resulting from alterations in the thermophoretic parameter leads to an increase in the thickness of the boundary layer. Furthermore, as the value of the thermophoretic parameter increases, there is a corresponding decline in the local Nusselt number, while the local Sherwood number exhibits an opposite trend.

**Figure 9:**
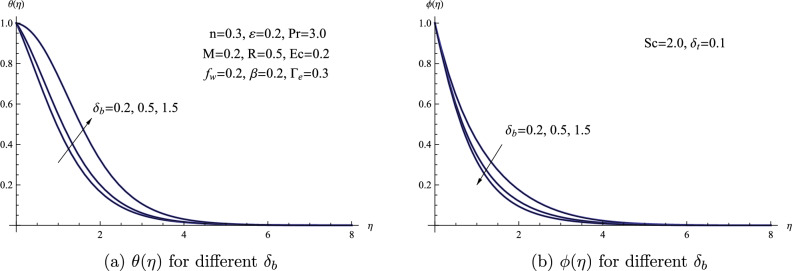
(**a**) $\theta (\eta )$ for different $\delta _{b}$ (**b**) $\phi (\eta )$ for different $\delta _{b}$.

Figure 9 portrays the concentration and temperature profiles for different values of Brownian motion parameter $\delta _{b}$. This graph clearly illustrates a direct correlation between the Brownian motion parameter and the profiles of both temperature and concentration. Elevating the Brownian motion parameter leads to a decrease in nanofluid concentration. This occurs because Brownian motion causes the particles to move away from the fluid region as the boundary layer experiences warming. Physically, an elevation in the Brownian motion parameter leads to increased thermal conduction as a result of the enhanced mobility of nanoparticles, thereby causing a rise in the temperature field.

**Figure 10:**
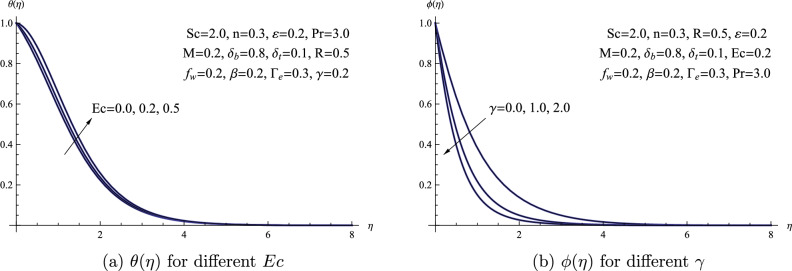
(**a**) $\theta (\eta )$ for different *Ec* (**b**) $\phi (\eta )$ for different $\gamma$.

The temperature profile $\theta (\eta )$ is depicted graphically in Fig. 10a in relation to variations in the Eckert number *Ec*. The correlation between the Eckert number *Ec* and the temperature distribution is highlighted by this visual representation. The relationship between the Eckert number and temperature differences is inverse; as the Eckert number increases, temperature differences become weaker, resulting in more pronounced temperature profiles and a thicker thermal boundary layer. Figure 10b illustrates the attributes of the chemical reaction parameter $\gamma$ and how it influences the distribution of concentration $\phi (\eta )$. A decrease in the concentration of the nanofluid results from increasing the chemical reaction parameter. This effect results from a drop in nanofluid concentration brought on by an enhanced chemical reaction parameter.

Table 2 presents the influences of various significant factors on key physical parameters, including the skin friction coefficient $Cf_{x}Re_{x}^{\frac{1}{2}}$, local Sherwood number $\frac{Sh_{x}}{\sqrt{Re_{x}}}$, and local Nusselt number $\frac{Nu_{x}}{\sqrt{Re_{x}}}$. This table offers a summary of how these diverse factors affect these critical physical quantities. As the power law index and the Weissenberg number increase, there is a corresponding decrease in the local Nusselt number, skin friction coefficient, and local Sherwood number. Also, the local Sherwood number, skin friction coefficient, and local Nusselt number all rise in response to a boost in the suction parameter. As the magnetic number is heightened, the skin friction coefficient experiences an increase. However, the local Sherwood number and the local Nusselt number decrease because of the Lorentz effect, which hinders fluid movement. As the value of the Brownian motion parameter rises, there is a concurrent decrease in the local Nusselt number, while the local Sherwood number experiences a simultaneous increase.

**Table 2:** Values of $Cf_{x}Re_{x}^{\frac{1}{2}}$, $\frac{Nu_{x}}{\sqrt{Re_{x}}}$ and $\frac{Sh_{x}}{\sqrt{Re_{x}}}$ for various values of $\Gamma _{e}, \beta , M, R, n, \varepsilon , \delta _{t}, \delta _{b}, Ec$ and $\delta _{b}$ with $Sc=2.0, \gamma =0.2$ and $Pr=3.0$.

$\Gamma _{e}$	$\beta$	*M*	$f_{w}$	*n*	*R*	$\varepsilon$	$\delta _{t}$	$\delta _{b}$	*Ec*	$Cf_{x}Re_{x}^{\frac{1}{2}}$	$\frac{Nu_{x}}{\sqrt{Re_{x}}}$	$\frac{Sh_{x}}{\sqrt{Re_{x}}}$
0.0	0.2	0.2	0.2	0.2	0.5	0.2	0.1	0.8	0.2	0.897563	0.197757	1.001520
1.0	0.2	0.2	0.2	0.2	0.5	0.2	0.1	0.8	0.2	0.852030	0.192528	0.989437
2.0	0.2	0.2	0.2	0.2	0.5	0.2	0.1	0.8	0.2	0.785905	0.183234	0.969761
0.5	0.0	0.2	0.2	0.2	0.5	0.2	0.1	0.8	0.2	0.779623	0.220553	1.012282
0.5	0.5	0.2	0.2	0.2	0.5	0.2	0.1	0.8	0.2	1.001912	0.162048	0.976054
0.5	1.0	0.2	0.2	0.2	0.5	0.2	0.1	0.8	0.2	1.178473	0.114776	0.950711
0.5	0.2	0.0	0.2	0.2	0.5	0.2	0.1	0.8	0.2	0.779623	0.220553	1.012282
0.5	0.2	0.5	0.2	0.2	0.5	0.2	0.1	0.8	0.2	1.001912	0.162048	0.976054
0.5	0.2	1.0	0.2	0.2	0.5	0.2	0.1	0.8	0.2	1.178473	0.114776	0.950711
0.5	0.2	0.2	0.0	0.2	0.5	0.2	0.1	0.8	0.2	0.805628	0.139805	0.830692
0.5	0.2	0.2	0.2	0.2	0.5	0.2	0.1	0.8	0.2	0.876164	0.195394	0.995976
0.5	0.2	0.2	0.5	0.2	0.5	0.2	0.1	0.8	0.2	0.991429	0.287496	1.267971
0.5	0.2	0.2	0.2	0.1	0.5	0.2	0.1	0.8	0.2	0.942036	0.199661	1.010632
0.5	0.2	0.2	0.2	0.3	0.5	0.2	0.1	0.8	0.2	0.822182	0.191896	0.983032
0.5	0.2	0.2	0.2	0.5	0.5	0.2	0.1	0.8	0.2	0.664586	0.175271	0.935341
0.5	0.2	0.2	0.2	0.3	0.0	0.2	0.1	0.8	0.2	0.822182	0.155684	0.989063
0.5	0.2	0.2	0.2	0.3	0.5	0.2	0.1	0.8	0.2	0.822182	0.191896	0.983032
0.5	0.2	0.2	0.2	0.3	1.0	0.2	0.1	0.8	0.2	0.822182	0.202175	0.980394
0.5	0.2	0.2	0.2	0.3	0.5	0.0	0.1	0.8	0.2	0.822182	0.189771	0.983851
0.5	0.2	0.2	0.2	0.3	0.5	0.5	0.1	0.8	0.2	0.822182	0.192263	0.982215
0.5	0.2	0.2	0.2	0.3	0.5	1.0	0.1	0.8	0.2	0.822182	0.198887	0.981486
0.5	0.2	0.2	0.2	0.3	0.5	0.2	0.0	0.8	0.2	0.822182	0.210753	0.982823
0.5	0.2	0.2	0.2	0.3	0.5	0.2	0.2	0.8	0.2	0.822182	0.174472	0.986165
0.5	0.2	0.2	0.2	0.3	0.5	0.2	0.5	0.8	0.2	0.822182	0.129694	1.009211
0.5	0.2	0.2	0.2	0.3	0.5	0.2	0.1	0.2	0.2	0.822182	0.460324	0.873602
0.5	0.2	0.2	0.2	0.3	0.5	0.2	0.1	0.5	0.2	0.822182	0.306576	0.964683
0.5	0.2	0.2	0.2	0.3	0.5	0.2	0.1	1.5	0.2	0.822182	0.037742	0.990519
0.5	0.2	0.2	0.2	0.3	0.5	0.2	0.1	0.8	0.0	0.822182	0.294832	0.971712
0.5	0.2	0.2	0.2	0.3	0.5	0.2	0.1	0.8	0.2	0.822182	0.191896	0.983032
0.5	0.2	0.2	0.2	0.3	0.5	0.2	0.1	0.8	0.5	0.822182	0.037095	1.000061

## Concluding remarks

This research offers a quantitative examination of the flow of a non-Newtonian tangent hyperbolic nanofluid over a surface that undergoes nonlinear stretching, while the flow takes place through a porous medium characterized by Darcy’s law. This study takes into account several factors, including chemical reactions, variable thermal conductivity, the magnetic field, the dissipation of viscous energy, and the influence of thermal radiation. After using the shooting technique to obtain the solution for our model, the numerical outcomes are presented in both tabular and graphical forms to emphasize the specific details of flow and heat transfer. Below is a compilation of the model’s most crucial findings.

1. The velocity profile is diminished by factors such as the porous parameter, magnetic number, suction parameter, power law index, and the Weisbsenberg number.
2. The skin friction coefficient exhibits a declining trend as the Weisbsenberg number and power law index rise, while it demonstrates an upward trend with increased values of the porous parameter, magnetic parameter, and suction parameter.
3. The nanofluid temperature experiences a decrease when the suction parameter and Prandtl number are increased, whereas it shows an increase as the Eckert number, Brownian motion parameter, radiation parameter, power law index, and magnetic number are increased.
4. Heat and mass transfer rates decline with an increase in the Weisbsenberg number, porous parameter, magnetic number, and power law index. Conversely, the suction parameter leads to the opposite effect, causing an increase in these rates.
5. The concentration distribution exhibits a decrease with an increase in both the Brownian motion parameter and suction parameter, whereas it shows an increase with higher values of the power law index and magnetic number.

## Data Availability

The datasets used and/or analyzed during the current study available from the corresponding author on reasonable request.
